# Basic Training in Palliative Medicine for Internal Medicine Residents: Pilot Testing of a Canadian Model in Switzerland

**DOI:** 10.1089/pmr.2024.0004

**Published:** 2024-04-15

**Authors:** Andreas Samuel Ebneter, Ebru Kaya, Petra Mair, Barbara Affollter, Steffen Eychmueller

**Affiliations:** ^1^ University Center for Palliative Care, University Hospital, Inselspital Bern, Switzerland.; ^2^ Division of Palliative Medicine, University of Toronto and University Health Network, Toronto, Ontario, Canada.; ^3^ Palliative Care Unit, Internal Medicine Clinic, Spital Thun, Thun, Switzerland.; ^4^ Palliative Care Unit, Internal Medicine Clinic, Spital Emmental, Burgdorf, Switzerland.

**Keywords:** curriculum, internal medicine, palliative care, palliative care unit, palliative medicine, postgraduate training

## Abstract

**Background:**

In Switzerland, palliative care (PC) clinical training is well established at undergraduate and specialist postgraduate levels. However, postgraduate nonspecialist training curricula are less documented.

**Local Problem:**

A structured curriculum for nonspecialist rotation within internal medicine (IM) in specialized PC wards is lacking.

**Objective:**

To pilot two versions of a PC nonspecialist curriculum for IM residents in Swiss PC units.

**Methods:**

In the pilot phase, two curricula—short immersion (3–10 weeks, based on the University of Toronto's Internal-Medicine PC Rotation) and standard nonspecialist (11–18 weeks, based on the Canadian Society of Palliative Care Physician Competencies)—were assessed using a mixed-method online survey. One university and two nonuniversity sites participated. The analysis was descriptive.

**Results:**

Five residents and eight supervisors of five training rotations (July–October 2023) responded. Overall, curriculum quality and feasibility (content and time) received positive ratings across all groups, with high satisfaction concerning organization, educational design, learning support, climate, experience, and facilities. Nonuniversity sites were generally rated more positively than university sites. Qualitative feedback paralleled these findings, highlighting the curriculum's relevance and fit with learners' needs and suggesting potential simplifications and more personalized planning.

**Conclusions:**

Establishing short and standard duration curricula for a PC program is viable and well received by nonspecialist trainees. Future implementation should concentrate on personalized learning objectives and streamlining the content and structure of the competencies. Cooperation within various training settings (university and regional hospitals) as well as on an international level (e.g., Canada–Switzerland) may further improve the quality of the proposed training formats.

## Introduction

Palliative care (PC) training equips physicians with the knowledge, skills, and attitudes necessary to meet diverse needs across general and specialist settings. All physicians must be skilled in providing generalist-level palliative medicine, which is distinct from the specialist level. In Switzerland, the undergraduate curriculum is well established.^1,2^ A checklist/competency catalogue exists for postgraduate learners in generalist palliative medicine, with the disadvantage of addressing a very broad range of trainees and having been developed 10 years ago.^3^

At the fellowship level, training for postgraduate specialists in palliative medicine is structured around clear frameworks, entailing three years of clinical training focused on attitude and skills and complemented by theoretical education to build knowledge, culminating in a summative examination.^4,5^ Numerous opportunities exist for professional development with a clear continuous medical education credit system.^6^

In Switzerland, palliative medicine is still evolving, and well-structured educational curricula are crucial for firmly establishing this relatively new medical specialty within the Swiss health care system.^7,8^ Despite voluntary basic training in palliative medicine,^9^ no structured curriculum for generalist palliative medicine currently exists as part of residencies in other specialties (i.e., internal medicine [IM]). This is significant because general PC is expected to address 80% of all PC situations, underlining the importance of well-structured and effective training for health care professionals in this field.

### Objective

The objective of this pilot phase was to evaluate the feasibility, acceptability, and appropriateness of the competencies of two new types of generalist palliative medicine rotations for IM residents: postgraduate immersion (PGI, 3–10 weeks) and postgraduate nonspecialist (PGNS, 11–18 weeks).

## Methods

### Design and study of the intervention

Thomas and Kern's framework^10^ informed this curriculum development project. This framework shares similarities of a quality improvement framework (Plan-Do-Act-Study), and consists of six steps: (1) problem identification and general needs assessment, (2) targeted needs assessment, (3) setting goals and objectives, (4) defining educational strategies, (5) implementation, and (6) evaluation.

A needs assessment (Thomas and Kern's steps 1 and 3) of the competency guidelines of the Canadian Society of Palliative Care Physicians,^11^ which preceded this pilot phase, is available as a conference abstract.^12^

For this pilot phase (Thomas and Kern's steps 4 and 5), we defined educational strategies, created a syllabus, and discussed these formally with the physician team. The syllabus included competencies, educational strategies, learning opportunities, and recommendations for workplace-based assessment (WBA) for two new curricula.

The PGNS curriculum (based on the competencies guidelines of the Canadian Society of Palliative Care Physicians (used with permission)^11^ and the previous targeted needs assessment.^12^

In addition, a shorter PGI curriculum based on the palliative medicine rotation of the University of Toronto^13^ (used with permission) was created to offer a structured curriculum to residents on short rotations.

Details of the final syllabi and the detailed competencies with proposed assessment structure are available in the Supplementary Appendix, including both the supervisor (Supplementary Appendices SA1-1 and SA2-1) and trainee (Supplementary Appendices SA1-2 and SA2-2) versions. An overview of the new educational/training curricula within the University Center for Palliative Care Inselspital (main site) training program is detailed in Table 1.

**Table 1.** Summary of Education/Training Curriculum Within the Training Program of the University Center for Palliative Care, Inselspital Bern, Switzerland

Curriculum	Framework/base	Duration
PGS	Swiss society for Palliative Care^4^	3 years
PGNS	Postgraduate competencies for palliative care (Canadian Society of palliative care physicians, used with permission)^11^	11–18 weeks (Usually 3–4 months)
PGI	Internal medicine–palliative care rotation (University of Toronto, used with permission)^13^	3–10 weeks (usually 3–6 weeks)
PCSD	SENS-Framework^14^ and local ACP framework: Iplan-Care^15^	<3 weeks
UGK	Palliative care objectives, University of Bern, individual goal setting according to the SENS Framework^14^	4 weeks

ACP, advanced care planning framework; PCSD, postgraduate palliative care service deployment; PGI, postgraduate-immersion; PGNS, postgraduate nonspecialist; PGS, postgraduate-specialist; UGK, undergraduate–clerkship.

After a brief (two-hour) faculty development session on competency-based medical education (CBME), WBA, and feedback,^16^ the pilot phase was implemented. The evaluation (Krickpatrick's level I: “learner reaction”^17^) used a mixed-method approach through a secure anonymous online survey (Lamapoll™).

The questionnaire had two parts: The first part was composed of (a) demographic data (differentiating between trainee and supervisor), (b) Likert scale queries (1–5, from very bad to very good) assessing overall quality and satisfaction with the educational organization, structure, and quality (adapted from the University of Toronto Rotation Evaluation, with permission), and (c) open-ended comments on strengths and improvement areas. The second part invited free-text feedback on the quality and relevant redundancies of the IM curriculum compared with the specified competencies of each curriculum (Supplementary Appendices S1-2 and S2-2).

The questionnaire was tested and reviewed by the authors, educational experts, and external supervising physicians. Analyses were descriptive, with quantitative results shown as median (range; interquartile range). The full questionnaire is available in its original language (German and machine translated in English) in Supplementary Appendices S3-1 and S3-2.

The provincial ethics board (Bern/Switzerland) confirmed this project as a quality improvement project (clarification of responsibility; Req-2023-00646).

### Context: Target population and setting

The target population for implementation and feedback was trainees (residents) as well as supervisors (PC unit attending physicans) in a convenience sample of training sites in the Swiss region of Bern. Three settings served as a pilot environment for the new curriculum: a university setting in a tertiary-level Swiss specialized PC unit and two nonuniversity settings in rural hospitals. All units participating in this pilot phase are subject to the same certification requirements;^18^ therefore, they are similar on a clinical level based on bed count. A detailed description of the setting is available in Supplementary Appendix S4.

## Results

### Summary of the preliminary work

The preliminary targeted needs assessment was a mixed method (relevance rating of the competencies, free text comments on quality, and relevance of the suggested competencies) online survey with the trainees (IM physicians). Results showed that the Canadian competencies were perceived as very suitable for a nonspecialist PC curriculum by the potential trainees and informed the definition of objectives and goals of the standard curriculum (PGNS).^12^

### Measurement of the pilot phase

Five training rotations—two PGNS-rotation and three PGI-rotation, split between university and nonuniversity settings—ran from July 2023 to October 2023. We received 13 valid responses (5 from trainees [100% response rate] and 8 from supervisors, 39% response rate), with 7 about the PGI-rotation and 6 about the PGNS-rotation. Residents had a median medical experience of three years (2–6), and nine years for supervisors (3–20). In total, 40% of residents self-declared that they had previous clinical PC experience. Median rotation durations were 4 weeks for PGI (2–5) and 12 weeks for PGNS (12–18).

The analysis of the data in an aggregated manner showed that curriculum quality was rated as good (4 [4–5;1]), with university centers rating it lower (4 [4–4;0]) than nonuniversity centers (5 [5–5;0]). Content feasibility was also deemed good (4–5;0). Time feasibility was rated good overall (4 [2–5;1]), but supervisors were more critical (median 3.5 vs. 4), especially for the longer PGNS curriculum (3.5 [2–4;1]) compared with the PGI (4 [3–5;1]). Nonuniversity centers rated time feasibility higher (4.5 [4–5;1]) than university centers (3 [2–4;1]).

The educational experience, overall organization, and facilities were rated good, with better ratings in nonuniversity settings. These results are detailed in Table 2.

**Table 2.** Summary of Results (Likert-Scale 1–5, Ascending-Symmetric, Very Bad–Average–Very Good, Cronbach's Alpha [All Items; 0.79])

Median (range; IQR)	Overall (***N*** = 13)	Trainee (***n*** = 5) vs. supervisors (***n*** = 8)		PGI (short) (***n*** = 7) vs. PGNS (long) curriculum (***n*** = 6)		University (***n*** = 9) vs. nonuniversity site (***n*** = 4)	
General quality	4 (4–5;1)	4 (4–5;1)	4 (4–5;1)	4 (4–5;1)	4 (4–5;1)	4 (4–4;0)	5 (5–5;0)
Feasibility content	4 (4–5;0)	4 (4–4;0)	4 (4–5;1)	4 (4–5;0)	4 (4–5;0)	4 (4–5;0)	4 (4–5;1)
Feasibility time	4 (2–5;1)	4 (3–5;1)	3.5 (2–5;1)	4 (3–5;2)	3.5 (2–4;1)	3 (2–4;1)	4.5 (4–5;1)
Organization of the site	4 (3–5;0)	4 (4–5;1)	4 (3–4;1)	4 (3–4;0)	4 (3–5;1)	4 (3–4;1)	4 (4–5;1)
Educational design	4 (3–5;1)	4 (3–5;1)	4 (4–5;1)	4 (4–5;1)	4 (3–5;1)	4 (3–5;0)	5 (4–5;1)
Educational support	4 (4–5;1)	4 (4–5;1)	4.5 (4–5;1)	4 (4–5;1)	4.5 (4–5;1)	4 (4–5;1)	5 (4–5;1)
Educational experience	4 (4–5;1)	5 (4–5;1)	4 (4–5;1)	4 (4–5;1)	5 (4–5;1)	4 (4–5;1)	5 (4–5;1)
Facilities	4 (3–5;0)	4 (4–5;1)	4 (3–5;1)	4 (3–5;1)	4 (4–5;0)	4 (3–5;1)	4 (4–5;1)

IQR, interquartile range.

The comparison of the reaction of the learners versus supervisors within the two curricula and the settings showed very similar ratings. Notably, time feasibility was rated lower by the supervisors at the university site compared with the trainees (4 [3–4;0] vs. 3 [2–4;1]). The results are detailed in Table 3

**Table 3.** Analysis of the Difference Between Trainees and Supervisors Between Curriculum and Settings (Likert-Scale 1–5, Ascending-Symmetric, Very Bad–Average–Very Good)

Median (range; IQR)	PGI (***n*** = 7)		PGNS (***n*** = 6)		University site (PGI/PGNS)		Nonuniversity site (PGI/PGNS)	
	Trainee (***n*** = 3)	Supervisor (***n*** = 4)	Trainee (***n*** = 2)	Supervisor (***n*** = 4)	Trainee (***n*** = 3)	Supervisor (***n*** = 6)	Trainee (***n*** = 2)	Supervisor (***n*** = 2)
General quality	4 (4–5;0)	4 (4–5;1)	4.5 (4–5;0)	4 (4–5;1)	4 (4–4;0)	4 (4–4;0)	5 (5–5;0)	5 (5–5;0)
Feasibility content	4 (4–4;0)	4 (4–5;1)	4 (4–4;0)	4 (4–5;1)	4 (4–4;0)	4 (4–5;0)	4 (4–4;0)	4.5 (4–5;0)
Feasibility time	4 (4–5;0)	3.5 (3–5;2)	3.5 (3–4;1)	3.5 (2–4;2)	4 (3–4;0)	3 (2–4;1)	4.5 (4–5;0)	4.5 (4–5;0)
Organization of the site	4 (4–4;0)	4 (3–4;1)	4.5 (4–1;0)	4 (3–4;1)	4 (4–4;0)	4 (3–4;1)	4.5 (4–5;0)	4 (4–4;0)
Educational design	4 (4–4;0)	4.5 (4–5;1)	4 (3–5;0)	4 (4–5;1)	4 (3–4;0)	4 (4–5;0)	4.5 (4–5;0)	5 (5–5;0)
Educational support	4 (4–4;0)	4.5 (4–5;1)	4.5 (4–5;0)	4.5 (4–5;1)	4 (4–4;0)	4 (4–5;1)	4.5 (4–5;0)	5 (5–5;0)
Educational experience	4 (4–5;0)	4 (4–5;1)	5 (5–5;0)	4.5 (4–5;1)	4 (4–5;0)	4 (4–5;1)	5 (5–5;0)	4.5 (4–5;0)
Facilities	4 (4–4;0)	3.5 (3–5;2)	4.5 (4–5;1)	4 (4–4;0)	4 (4–5;0)	4 (3–4;1)	4 (4–4;0)	4.5 (4–5;0)

Free text feedback emphasized that the competencies aligned well with the clinical needs and the IM curriculum. Strengths noted include comprehensive PC objectives, improved feedback, and increased motivation for learning and teaching. Recommendations for enhancement included more formal scheduling, simplified documentation, and a resident-led content priority setting.

Regarding PGI-Curriculum-Competencies, the main concern was potential overlap with IM skills, with no specific omissions reported. Feedback on PGNS competencies included time efficiency concerns and a disproportionate focus on spiritual/social aspects, with end-of-life communication noted as a gap. Examples of free text are shown in Supplementary Appendix S5.

## Discussion

The pilot phase of two different formats of embedded nonspecialist PC curricula for IM residents in a postgraduate PC training program demonstrated strong feasibility and acceptability, aligning well with clinical training needs. The adaptation of existing competencies to a postgraduate, short, immersion, and standard duration, nonspecialist curricula proved effective.

Both curricula showed high feasibility and satisfaction, noteworthy in Switzerland's transition toward a CBME model. The positive reception was aided by a preliminary detailed needs assessment,^12^ preparatory faculty development,^16^ and application of established competencies.^11^ Nonuniversity centers particularly benefited, likely due to more direct trainee–supervisor interactions.^19^

The results of our pilot phase showed a good time feasibility overall and the absence of negative free text feedback about WBA.

This is notable because competence-based educational models require more time for the faculty, mainly due to the time-consuming (WBA), which is known to be a limiting factor for curriculum development.^20^ In our program, we limited the assessment types to three (structured feedback,^21^ case-based discussions, and Mini-CEX^22^). Therefore, embedding these WBAs in a well-structured curriculum may make them feasible within available time resources.

Qualitative feedback affirmed the competencies' alignment with learner needs in IM, underscoring the need assessment's value.

The positive results were encouraging, especially because of the initially apprehended barriers, which were (a) the amount of change with respect to the usual training models (time-based educational model), (b) adding complexity and administrational burden to the workday of the residents and supervisors), and (c) acceptance within the faculty. The strategy to overcome these was a highly participatory learner and supervisor needs-focused approach, which emphasized the advantages of having such a curriculum (e.g., structured training, increased feedback quality, and longer rotation time with better continuity).

Based on the results of the pilot phase, the educational experience can be improved by simplifying the documentation process and, more crucially, ensuring that the competencies are concise and customizable. Future adaptations will prioritize participatory selection of competencies over mere completion, enhancing the breadth of the PGI's 9 competencies and the clarity and focus of the PGNS's 52 specific competencies.

This pilot phase's strengths include its seamless integration into the existing curriculum and its ability to encompass both university and nonuniversity settings. Its limitations, namely the small participant pool, are offset by the pilot phase's targeted focus as a quality improvement initiative, although this did restrict comparative analysis between settings.

This was a local curriculum/quality improvement project, with a small number of participants. Therefore, transferability to other settings remains open. Nonetheless, we believe that the results of the pilot phase can serve as an example of feasibility and acceptance of such types of curriculums. If adapted to another setting, this should be done in close collaboration with both trainees and faculty and with a clear evaluations strategy.

In conclusion, the pilot phase's outcomes support the integration of the two curricula into our framework, providing insights for enhancing clarity and efficiency in the PC training program. The next step would be a larger scale implementation with an evaluation at least at Kirkpatrick's level 2 (learning effect).

## Supplementary Material

Supplemental data

Supplemental data

Supplemental data

Supplemental data

Supplemental data

Supplemental data

Supplemental data

Supplemental data

## Supplementary Material

Supplementary Appendix SA1-1

Supplementary Appendix SA1-2

Supplementary Appendix SA2-1

Supplementary Appendix SA2-2

Supplementary Appendix SA3-1

Supplementary Appendix SA3-2

Supplementary Appendix SA4

Supplementary Appendix SA5

## Abbreviations Used

ACP: advanced care planning framework
CBME: competency-based medical education
IM: internal medicine
PC: palliative care
PCSD: postgraduate-palliative care-service deployment
PGI: postgraduate immersion
PGNS: postgraduate nonspecialist
PGS: postgraduate specialist
UGK: undergraduate–clerkship
WBA: workplace-based assessment
