# Supplementary material for: Basic Training in Palliative Medicine for Internal Medicine Residents: Pilot Testing of a Canadian Model in Switzerland
Source: Palliat Med Rep. 2024 Apr 15;5(1):171–6. doi: 10.1089/pmr.2024.0004 (PMC11043622; doi:10.1089/pmr.2024.0004)
Supplement: Supplemental data [file Suppl_AppSA2-1.pdf]

"River-Aare-Curriculum-University Center for Palliative Care, Inselspital, Bern
Palliative Care Postgraduate non-specialist curriculum (PGNS) for internal medicine residents (2.5 month - 4.5 month)

Based on "postgraduate competencies for palliative care - a guidance document" (Herx L, Gofton TE, Bromwich C, et al. Postgraduate Competencies for Palliative Care - a Guidance Document. Guidance Document. CSPCP; 2019.) Used with permisson from the Canadian Society of Palliative Medicine for the River Aare-Curriculum (University Center for Palliative Care, Inselspital Bern, Bern,Switzerland). Any use other needs approval by the Canadian Societey of Palliative Medicine.

CANMED	Competency	Enabling competencies	Specific competencies	Learning opportunities	Learning strategies
Medical Expert	Provide a palliative approach to care				
		Identify when to initiate a palliative approach to care in all settings.		clinic	
			Recognize common trajectories, natural histories of functional decline, and transition points to trigger early initiation of a palliative approach to care.	clinic/weekly patient review	text-based/small group discussions
			Describe the benefits of an early palliative approach to care.	clinic/daily report	text-based/peer-teaching
	Assess and manage pain and other common symptoms in their patient populations.				
		Assess and manage pain in a palliative context.			
			Conduct a thorough pain history and perform an appropriate physical exam for a patient presenting with pain	clinic	supervised clinicial experience
			Demonstrate a patient- and family-centered and interprofessional approach to assessing pain in patients with life-threatening illness.	clinic	supervised clinicial experience
			Describe and recognize "total pain", where physical, psychological, social, emotional and spiritual concerns each contribute to the pain experience.	clinic	peer-teaching
			Describe and use standardized tools for pain assessment.	clinic/self learning	text based
			Choose appropriate/relevant investigations of pain	clinic	supervised clinicial experience
			Choose an appropriate analgesia regimen, including non-pharmacological and pharmacological elements.	clinic/self learning	text based/supervised clinical experience
		Use opioids effectively to manage pain and other symptoms in a palliative context.			
			Write an appropriate opioid prescription for an opioid-naïve patient, including breakthrough dosing.	clinic	supervised clinicial experience
			Manage common routes of opioid administration and their effect on bioavailability and dosing frequency.	clinic	peer-teaching/supervised clinical experience
			Manage relevant pharmacokinetic and pharmacodynamics properties of opioids, including patient-specific considerations such as age, weight, prior exposure and renal and hepatic function.	clinic	supervised clinicial experience
			Demonstrate appropriate opioid titration.	clinic	supervised clinicial experience
			Manage common side effects of opioids and anticipate and prevent side effects such as nausea and constipation.	clinical	supervised clinicial experience
			Address patient and family concerns or myths about opioids.	clinical/self learning	peer-teaching
			Explain the concepts of tolerance, physical dependence and addiction as they relate to the use of opioids.	clinical	peer-teaching
			Identify potential risk factors for opioid misuse, abuse, addiction and/or diversion, and describe approaches for managing these issues.	clinical	peer-teaching
			Recognize opioid-induced neurotoxicity (OIN) and distinguish OIN from opioid overdose.	clinicial	peer-teaching
		Use adjuvant modalities and medications for pain management in a palliative context.			
			Use adjuvant analgesics appropriately, including but not limited to corticosteroids, nonsteroidal anti-inflammatory drugs and neuropathic agents.	clinical/daily visit	text-based, small group discussion
			Recognize the potential role for adjuvant modalities, including but not limited to chemotherapy, radiation therapy, surgery and interventional analgesia in the management of pain and other symptoms, and refer when appropriate.	clinical	peer-teaching
		Assess and manage common symptoms, including but not limited to constipation, nausea and vomiting, dyspnea, delirium, and insomnia.			
			Conduct a thorough history and perform an appropriate physical exam for a patient presenting with common symptoms.	clinical	supervised clinical experience
			Demonstrate a patient- and family-centered and interprofessional approach to assessing symptoms in patients with life-threatening illness.	clinical	supervised clinical experience
			Describe and use validated tools for symptom assessment as appropriate for the patient population.	clinical	supervised clinical experience
			Choose appropriate/relevant investigations for identified symptoms.	clinical	supervised clinical experience
			Initiate appropriate first-line therapy to manage identified symptoms, including nonpharmacological and pharmacological interventions.	clinical	supervised clinical experience
		Appropriately assess and describe the elements of suffering for patients receiving a palliative approach to care and their families.			
			Integrate diverse societal perspectives on dying and death	clinical	peer-teaching
			Identify and describe issues contributing to suffering in patients requiring a palliative approach to care and their families	clinical	peer-teaching
		Provide a supportive approach to suffering.			
			Demonstrate a supportive approach to address multidimensional sources of suffering in patients with palliative care needs and their families.	clinical	exposure/reflection
	Participate in the care of the dying patient and their family in uncomplicated cases.				
		Participate in the care of the dying patient and their family in uncomplicated cases.			
			Identify signs of approaching death.	clinical	text-based/peer-teaching
			Describe common signs of the natural dying process.	clinical	text-based/peer-teaching
			Prepare and educate the patient and family when death approaches.	clinical	supervised clinical experience
			Prescribe medications for symptom control in the dying phase.	clinical	supervised clinical experience
			Pronounce a patient's death and complete a death certificate.	clincial	demonstration
	Participate in providing care for the child requiring a palliative approach to care and their family, if provision of pediatric care is applicable to scope of practice.	not applicable in Switzerland = Domain of pediatrics			
	Address psychosocial and spiritual needs of patients requiring a palliative approach to care and their families.				
		Address psychosocial and spiritual issues that patients with life-threatening illness and their families encounter.			
			Identify, assess and plan for the psychosocial and spiritual needs that patients and their families encounter across the illness trajectory.	clinical	supervised clinical experience
			Recognize the level of demand and stress of caregivers and identify risk factors for caregiver burnout.	clinical	peer-teaching
			Demonstrate the ability to screen, diagnose and initiate treatment for patients experiencing depression and/or anxiety.	clinical	supervised clinical experience
			Identify risk factors for complicated grief.	clinical/self learning	peer-teaching
			Identify patients and caregivers who have complex psychosocial needs who would benefit from referral to expert resources.	clinical	peer-teaching
			Describe the relationship between psychosocial, spiritual and cultural issues with respect to total suffering and total pain.	clinical	peer-teaching
			Describe an approach to provide or refer for supportive care for someone experiencing anticipatory grief and someone experiencing bereavement.	clinical	peer-teaching
		Develop and propose a care plan in collaboration with other disciplines .			
			Collaborate in the development of an interprofessional care plan to meet the psychosocial and spiritual needs of patients and families facing life-threatening illness.	clinical	exposure/supervised clinical experience)
			Actively involve primary care providers and other community-based supports in the psychosocial and spiritual support of patients and families facing life-threatening illness.	clinical	exposure/reflection
		Create an environment of cultural safety, demonstrating sensitivity to spiritual, religious and cultural considerations, and to life context.			
			Demonstrate cultural humility and describe how diversity impacts decision making, to provide patient and family centered care.	clincial	role-modeling
			Recognize when the values, biases or perspectives of patients, physicians or other health care professionals may have an impact on the quality of care and modify the approach to the patient and family accordingly.	clincial	role-modeling

Copyright: Andreas .Ebneter, MME-Candidate and member of the UZP Team. (andreassamuel.ebneter@insel.ch)
Developed as a MME-Projet (MME-Unibe)

Leader and Professional	Actively engage in advance care planning, goals of care and decision making with patients who would benefit from a palliative approach to care, using bioethical and legal frameworks.				
		Establish advance care plans with patients and families in accordance with provincial / territorial regulations and terminology.			
			Discuss the common ethical issues that arise throughout the illness trajectory such as decision making, withdrawing or withholding therapy, and resuscitation orders.	clinical	oral reflection
			Demonstrate respect for differing family structures, roles, and cultural issues when sharing information and arriving at decisions, including care plans.	clinical	exposure
		Demonstrate the use of advance care planning.	Demonstrate an effective approach to advance care planning.	clinical	text-based/demonstration
		Distinguish between Medical Assistance in Dying (MAiD), palliative sedation, withholding or withdrawing therapy in accordance with provincial/territorial/federal regulations and terminology.			
			Demonstrate the ability to respond to patients and families when discussing MAiD, palliative sedation and withholding or withdrawing therapy.	clinical	text-based/small group discussion
			Compassionately explore and address patient and family suffering in these contexts.	clinical	supervised clinical experience
			Involve specialized palliative care services when appropriate.	clinical	peer teaching
		Demonstrate self-reflection and self-care in working with patients requiring a palliative approach to care and their families.			
			Identify common factors contributing to personal and professional stress in caring for patients who require a palliative approach to care and their families.	clinical	peer teaching
			Develop a plan to cope with personal and professional stress that may arise in caring for patients who require a palliative approach to care and their families.	clinical	reflection
			Exhibit self-reflective capacity in analyzing one's own values, beliefs and reactions when faced with dying and death.	clinical	reflection
			Demonstrate awareness when personal reactions may impact the ability to provide a palliative approach to care and seek help to mitigate.	clinical	reflection
Communicator	Communicate effectively with patients, families and other informal caregivers.				
		Communicate honestly and compassionately about life-threatening illness and prognosis from the time of diagnosis and throughout the illness trajectory.			
			Elicit the patient's and family's understanding of their illness and readiness for information sharing.	clinical	supervised clinical practice /role-play
			Demonstrate the ability to discuss an individualized estimation of survival and disease trajectory.	clinical	supervised clinical practice /role-play
		Independently facilitate patient and family meetings.		clinical	demonstration/supervised clinical practice
		Communicate with patients and families in order to determine, record, and implement a care plan aligned with the patient's values and goals of care.		clinical	supervised clinical practice
		Demonstrate the ability to educate patients and families receiving a palliative approach to care about matters related to advancing disease.		clinical	supervised clinical practice
		Communicate with health care providers, including the primary care team, about the natural history of the illness, what to monitor, when to refer, prognostication, and suggestions around "community-based action plans".		clinical	exposure
Collaborator	Collaborate as members of an interprofessional team.				
		Work effectively with interprofessional colleagues to provide a palliative approach to care throughout the illness trajectory.		clinical	supervised clinical experience
		Make effective referrals for patients with complex needs requiring specialized palliative care expertise; including but not limited to: reasons for consultation, pertinent investigations, pain management, medication list, opioid toxicity.		clinical	supervised clinical experience
		Ensure the continuity of a palliative approach to care across different settings by collaborating with the most responsible clinician.		clinical	supervised clinical experience
		Demonstrate the ability to collaborate with other disciplines regarding which serious illness conversations have occurred and share the patient and family's reactions.		clinical	supervised clinical experience
Health Advocate	Identify determinants of health and address barriers impacting palliative care provision for an individual patient and the population served.				
		Identify and, where possible, address barriers for availability and accessibility of palliative care, including but not limited to: geography, stigma associated with receiving palliative care, lack of recognition of people who would benefit, availability of community resources, availability of specialized palliative care services.		clinical/weekly staff meetings	peer teaching/group discussion
		Identify and work in partnership with allies among vulnerable and marginalized populations to address the inequities in their access to palliative care, including but not limited to: homeless, Indigenous peoples, incarcerated individuals and those in rural/remote communities.		clinical/weekly staff meetings	peer teaching/group discussion

Copyright: Andreas .Ebneter, MME-Candidate and member of the UZP Team. (andreassamuel.ebneter@insel.ch)
Developed as a MME-Projet (MME-Unibe)
