# Supplementary material for: Basic Training in Palliative Medicine for Internal Medicine Residents: Pilot Testing of a Canadian Model in Switzerland
Source: Palliat Med Rep. 2024 Apr 15;5(1):171–6. doi: 10.1089/pmr.2024.0004 (PMC11043622; doi:10.1089/pmr.2024.0004)
Supplement: Supplemental data [file Suppl_AppSA1-2.pdf]

River-Aare-Curriculum-University Center for palliative Care, Inselspital, Bern
Palliative Care Postgraduate immersion Curriculum (PGI) for internal medicine residents (3- 12 weeks)

			OPTIONAL Workplace-based Assessements	
	Individual goals (resident, selfrating)	Assessment/Discussion Structured Feedback (SFED-Framework) with/from Supervisor	Case based discussion	Mini-CEX
**Competencies**	**c = confident at start; x = general, actual priority; text... = specific goal**	**Date**	**Date**	**date**
Performs a comprehensive palliative care history, including assessing history, physical, social, psychological, spiritual, and functional parameters. Based on the SENS-Framework				
Formulates an appropriate plan for the investigation and treatment (including pharmacological and non-pharmacological treatments) for common symptoms in palliative care patients.				
Describes a patient-centred, evidence-based approach to the management of patients requiring palliative care.				
Understands unique aspects of pharmacotherapy in the patient requiring palliative care.				
Demonstrates an ability to work with the patient and family to establish patient centred goals of care. understand the distinction between MAID and palliative care.				
Participate in interdisciplinary care of patients, including family meetings.				
Collaborate appropriately with other MRP, consulting and allied health care teams involved in the patient’s care when formulating a care plan.				
Identify and engage in opportunities to optimize alignment of care plan and patient’s goals and values based on the local Iplan Framework				
Selects palliative care resources available for disposition planning.				

Copyright notice: This document is based on the Internal Medicine ITAR for Palliative Care, PGY2, University of Toronto, and subject to copyright and used with permission. Any other use than in the context of the River-Aare-Curriculum from the Palliative Care Center of the Inselspital Bern, has to be validated/requested by/at the head of the Palliative Care program at the University Toronto (Prof. Ebru Kaya)
