# Supplementary material for: Basic Training in Palliative Medicine for Internal Medicine Residents: Pilot Testing of a Canadian Model in Switzerland
Source: Palliat Med Rep. 2024 Apr 15;5(1):171–6. doi: 10.1089/pmr.2024.0004 (PMC11043622; doi:10.1089/pmr.2024.0004)
Supplement: Supplemental data [file Suppl_AppSA2-2.pdf]

"River-Aare-Curriculum-University Center for Palliative Care, Inselspital, Bern
Palliative Care Postgraduate non-specialist curriculum (PGNS) for internal medicine residents (2.5 month - 4.5 month)

Based on "postgraduate competencies for palliative care - a guidance document" (Herx L, Gofton TE, Bromwich C, et al. Postgraduate Competencies for Palliative Care - a Guidance Document. Guidance Document. CSPCP; 2019.) Used with permisson from the Canadian Society of Palliative Medicine for the River Aare-Curriculum (University Center for Palliative Care, Inselspital Bern, Bern,Switzerland). Any other use needs approval by the Canadian Society of Palliative Medicine. (office@cspcp.ca)

CANMED	Competency	Enabling competencies	Specific competencies	Individual goals (resident)<br>"c" = confident at start<br>"x" = current priority<br>"free text" = specific	Feedback/Discussion with/from Supervisor	Workplace-based Assessements	
						Case based discussion	Mini-CEX/DOPS
	Provide a palliative approach to care	Identify when to initiate a palliative approach to care in all settings.	Recognize common trajectories, natural histories of functional decline, and transition points to trigger early initiation of a palliative approach to care.<br>Describe the benefits of an early palliative approach to care.				
	Assess and manage pain and other common symptoms in their patient populations.	Assess and manage pain in a palliative context.	Conduct a thorough pain history and perform an appropriate physical exam for a patient presenting with pain<br>Demonstrate a patient- and family-centered and interprofessional approach to assessing pain in patients with life-threatening illness.<br>Describe and recognize "total pain", where physical, psychological, social, emotional and spiritual concerns each contribute to the pain experience.<br>Describe and use standardized tools for pain assessment.<br>Choose appropriate/relevant investigations of pain<br>Choose an appropriate analgesia regimen, including non-pharmacological and pharmacological elements.				
		Use opioids effectively to manage pain and other symptoms in a palliative context.	Write an appropriate opioid prescription for an opioid-naïve patient, including breakthrough dosing.<br>Manage common routes of opioid administration and their effect on bioavailability and dosing frequency.<br>Manage relevant pharmacokinetic and pharmacodynamics properties of opioids, including patient-specific considerations such as age, weight, prior exposure and renal and hepatic function.<br>Demonstrate appropriate opioid titration.<br>Manage common side effects of opioids and anticipate and prevent side effects such as nausea and constipation.<br>Address patient and family concerns or myths about opioids.<br>Explain the concepts of tolerance, physical dependence and addiction as they relate to the use of opioids.<br>Identify potential risk factors for opioid misuse, abuse, addiction and/or diversion, and describe approaches for managing these issues.<br>Recognize opioid-induced neurotoxicity (OIN) and distinguish OIN from opioid overdose.				
		Use adjuvant modalities and medications for pain management in a palliative context.	Use adjuvant analgesics appropriately, including but not limited to corticosteroids, nonsteroidal anti-inflammatory drugs and neuropathic agents.<br>Recognize the potential role for adjuvant modalities, including but not limited to chemotherapy, radiation therapy, surgery and interventional analgesia in the management of pain and other symptoms, and refer when appropriate.				
		Assess and manage common symptoms, including but not limited to constipation, nausea and vomiting, dyspnea, delirium, and insomnia.	Conduct a thorough history and perform an appropriate physical exam for a patient presenting with common symptoms.<br>Demonstrate a patient- and family-centered and interprofessional approach to assessing symptoms in patients with life-threatening illness.<br>Describe and use validated tools for symptom assessment as appropriate for the patient population.<br>Choose appropriate/relevant investigations for identified symptoms.<br>Initiate appropriate first-line therapy to manage identified symptoms, including nonpharmacological and pharmacological interventions.				
Medical Expert		Appropriately assess and describe the elements of suffering for patients receiving a palliative approach to care and their families.	Integrate diverse societal perspectives on dying and death<br>Identify and describe issues contributing to suffering in patients requiring a palliative approach to care and their families				
		Provide a supportive approach to suffering.	Demonstrate a supportive approach to address multidimensional sources of suffering in patients with palliative care needs and their families.				
	Participate in the care of the dying patient and their family in uncomplicated cases.	Participate in the care of the dying patient and their family in uncomplicated cases.	Identify signs of approaching death.<br>Describe common signs of the natural dying process.<br>Prepare and educate the patient and family when death approaches.<br>Prescribe medications for symptom control in the dying phase.<br>Pronounce a patient's death and complete a death certificate.				
	Participate in providing care for the child requiring a palliative approach to care and their family, if provision of pediatric care is applicable to scope of practice.	not applicable in Switzerland = Domain of pediatrics					
	Address psychosocial and spiritual needs of patients requiring a palliative approach to care and their families.	Address psychosocial and spiritual issues that patients with life-threatening illness and their families encounter.	Identify, assess and plan for the psychosocial and spiritual needs that patients and their families encounter across the illness trajectory.<br>Recognize the level of demand and stress of caregivers and identify risk factors for caregiver burnout.<br>Demonstrate the ability to screen, diagnose and initiate treatment for patients experiencing depression and/or anxiety.<br>Identify risk factors for complicated grief.<br>Identify patients and caregivers who have complex psychosocial needs who would benefit from referral to expert resources.<br>Describe the relationship between psychosocial, spiritual and cultural issues with respect to total suffering and total pain.<br>Describe an approach to provide or refer for supportive care for someone experiencing anticipatory grief and someone experiencing bereavement.				
		Develop and propose a care plan in collaboration with other disciplines .	Collaborate in the development of an interprofessional care plan to meet the psychosocial and spiritual needs of patients and families facing life-threatening illness.<br>Actively involve primary care providers and other community-based supports in the psychosocial and spiritual support of patients and families facing life-threatening illness.				
		Create an environment of cultural safety, demonstrating sensitivity to spiritual, religious and cultural considerations, and to life context.	Demonstrate cultural humility and describe how diversity impacts decision making, to provide patient and family centered care.<br>Recognize when the values, biases or perspectives of patients, physicians or other health care professionals may have an impact on the quality of care and modify the approach to the patient and family accordingly.				
	Actively engage in advance care planning, goals of care and decision making with patients who would benefit from a palliative approach to care, using bioethical and legal frameworks.	Establish advance care plans with patients and families in accordance with provincial / territorial regulations and terminology.	Discuss the common ethical issues that arise throughout the illness trajectory such as decision making, withdrawing or withholding therapy, and resuscitation orders.<br>Demonstrate respect for differing family structures, roles, and cultural issues when sharing information and arriving at decisions, including care plans.				
		Demonstrate the use of advance care planning.	Demonstrate an effective approach to advance care planning.				
Leader and Professiona		Distinguish between Medical Assistance in Dying (MAiD), palliative sedation, withholding or withdrawing therapy in accordance with provincial/territorial/federal regulations and terminology.	Demonstrate the ability to respond to patients and families when discussing MAiD, palliative sedation and withholding or withdrawing therapy.<br>Compassionately explore and address patient and family suffering in these contexts.<br>Involve specialized palliative care services when appropriate.				
		Demonstrate self-reflection and self-care in working with patients requiring a palliative approach to care and their families.	Identify common factors contributing to personal and professional stress in caring for patients who require a palliative approach to care and their families.<br>Develop a plan to cope with personal and professional stress that may arise in caring for patients who require a palliative approach to care and their families.<br>Exhibit self-reflective capacity in analyzing one's own values, beliefs and reactions when faced with dying and death.<br>Demonstrate awareness when personal reactions may impact the ability to provide a palliative approach to care and seek help to mitigate.				
Communicator	Communicate effectively with patients, families and other informal caregivers.	Communicate honestly and compassionately about life-threatening illness and prognosis from the time of diagnosis and throughout the illness trajectory.<br>Independently facilitate patient and family meetings.<br>Communicate with patients and families in order to determine, record, and implement a care plan aligned with the patient's values and goals of care.<br>Demonstrate the ability to educate patients and families receiving a palliative approach to care about matters related to advancing disease.<br>Communicate with health care providers, including the primary care team, about the natural history of the illness, what to monitor, when to refer, prognostication, and suggestions around "community-based action plans".	Elicit the patient's and family's understanding of their illness and readiness for information sharing.<br>Demonstrate the ability to discuss an individualized estimation of survival and disease trajectory.				
Collaborator	Collaborate as members of an interprofessional team.	Work effectively with interprofessional colleagues to provide a palliative approach to care throughout the illness trajectory.<br>Make effective referrals for patients with complex needs requiring specialized palliative care expertise; including but not limited to: reasons for consultation, pertinent investigations, pain management, medication list, opioid toxicity.<br>Ensure the continuity of a palliative approach to care across different settings by collaborating with the most responsible clinician.<br>Demonstrate the ability to collaborate with other disciplines regarding which serious illness conversations have occurred and share the patient and family's reactions.					
Health Advocate	Identify determinants of health and address barriers impacting palliative care provision for an individual patient and the population served.	Identify and, where possible, address barriers for availability and accessibility of palliative care, including but not limited to: geography, stigma associated with receiving palliative care, lack of recognition of people who would benefit, availability of community resources, availability of specialized palliative care services.<br>Identify and work in partnership with allies among vulnerable and marginalized populations to address the inequities in their access to palliative care, including but not limited to: homeless, Indigenous peoples, incarcerated individuals and those in rural/remote communities.					
