# Supplementary material for: Basic Training in Palliative Medicine for Internal Medicine Residents: Pilot Testing of a Canadian Model in Switzerland
Source: Palliat Med Rep. 2024 Apr 15;5(1):171–6. doi: 10.1089/pmr.2024.0004 (PMC11043622; doi:10.1089/pmr.2024.0004)
Supplement: Supplemental data [file Suppl_AppSA3-1.pdf]

Evaluation der Pilot-Phase des "River-Aare-Curriculums"

Sehr geehrte Kolleginnen und Kollegen

Wir möchten Ihnen zuerst für die Teilnahme an der Pilotphase des River-Aare-Curriculums auf unserer Station danken. Wir hoffen, dass etwas vom Gelernten für Ihre zukünftige Laufbahn hilfreich sind wird.
Zur Verbesserung und Anpassung der Curriculum an die Bedürfnisse von Internist_innen möchten auch wir von Ihnen lernen. Deshalb erhalten Sie heute von uns einen Fragebogen. Dieser ist kurz gehalten und die Dauer für das Aufüllen beträgt ca. 10-15 min.

Es würde uns sehr freuen, wenn wir eurer Rückmeldungen haben könnten und danken euch schon im Voraus für euere Interesse.

Andreas Ebneter, für das Projekt Team

Projektteam:
- Andreas Ebneter; Steffen Eychmüller; Petra Mair; Barbara Affolter

Copyright: Das Projekt basiert auf einem Curriculum der Canadian Society of Palliative Care Physicians (CSPCP) und der University of Toronto. Der Inhalt unterliegt einem Copyright und darf nicht ohne Einverständnis weitergeben/geteilt werden. Wir danken unseren Kollegen, insbesondere unserer internationalen Beraterin (Prof. Ebru Kaya), für die Erlaubnis, es zu verwenden und an den Schweizer Kontext anzupassen.

Datenschutz: Ihre Daten werden anonymisiert (bei Eingabe ins Online-Tool), verschlüsselt und ohne Rückschlüsse auf Einzelpersonen analysiert. Mit dem Beantworten dieser Umfrage erklären Sie sich einverstanden, dass Ihre Antworten (anonymisiert) für eine Publikation verwendet werden dürfen.

Ethik/Datenschutz: Die Umfrage ist Teil einer Qualitätsverbesserungs-Begleitstudie, die kantonale Ethikkommission hat den Projektplan gesichtet und als nicht-genehmigungspflichtig bestätigt (Qualitätssicherungs-Studie,Req-2023-00646), die Datenschutzregeln werden gemäss GCP eingehalten, das online Umfrage-Tool ist gemäss DSGVO (Europa) zertifiziert, die Daten werden in Deutschland gespeichert)

Sind Sie mit der Teilnahme einverstanden ?

- ◯ Ja, ich mache gerne mit
- ◯ Nein, lieber nicht

INSELSPITAL
UNIVERSITÄTSSPITAL BERN
HÔPITAL UNIVERSITAIRE DE BERNE

UNIVERSITÄRES ZENTRU
FÜR PALLIATIVE CARE

Evaluation des "River-Aare-Curriculums"

Meine Rolle in der Klinik ist...

- ◯ Assistenzärztin/Assistenzarzt (Trainee)
- ◯ Ausbildner:in (Supervisor)

Meine Arbeitsort

◯ Universitäts-Spital ◯ Nicht-universitäres Spital

Wie viel Jahre Erfahrung haben Sie als Ärztin/Arzt

[ ] Jahre

Haben Sie schon klinische Erfahrung in der Palliative Care ??

◯ Ja, folgende Anzahl Monate [ ] ◯ Nein

Wie sicher fühlen Sie sich Patient:innen mit Palliative Care Bedürfnissen zu betreuen

unsicher	eher unsicher	weder sicher/noch unsicher	eher sicher	sicher
○	○	○	○	○

Welche Curriculum-Struktur haben Sie während Ihrer Rotation auf der Palliativstation absolviert ?

○ "Langes" Curriculum ○ "kurzes" Curriculum

Wie viele Wochen waren Sie auf der Palliativstation

[ ] Wochen

Auf welche Curriculumstruktur bezieht sich ihre Einschätzung als Supervisor:in ?

○ "Langes" Curriculum ○ "kurzes" Curriculum

Beurteilen Sie, als Supervisor:in das erste Mal das River-Aare-Curriculum ?

○ Ja

○ Nein, (bitte Anzahl der vorangehenden Evaluationen eingeben) [ ]

Wie beurteilen Sie die allgemeine Qualität des River-Aare-Curriculum

sehr schlecht	schlecht	durchschnittlich	gut	sehr gut
○	○	○	○	○

Wie beurteilen Sie die Machbarkeit des River-Aare-Curriculum?

	sehr schlecht	schlecht	durchschnittlich	gut	sehr gut
Hinsichtlich Umfangs/Menge der Lerninhalte	○	○	○	○	○
Hinsichtlich zeitlichen Ressourcen	○	○	○	○	○

Wie beurteilen Sie die folgenden Aspekte des River-Aare-Curriculum

	sehr schlecht	schlecht	durchschnittlich	gut	sehr gut
Organisation	○	○	○	○	○
Pädagogische Gestaltung	○	○	○	○	○
Unterstützung des Lernprozesses	○	○	○	○	○
Lern-/Lehrerfahrung	○	○	○	○	○
Austattung/Räumlichkeiten	○	○	○	○	○

INSELSPITAL
UNIVERSITÄTSSPITAL BERN
HÔPITAL UNIVERSITAIRE DE BERNE

UNIVERSITÄRES ZENTRU
FÜR PALLIATIVE CARE

Qualitative Kommentare

Im nächsten Abschnitt haben Sie die Möglichkeit uns individuelle Kommentare zukommen zu lassen. Diese dürfen auch kurz (Stichwortartig) sein.

Kommentare zur allgemeinen Qualität/Praxisrelevanz des River-Aare-Curriculums

Bitte beschreiben Sie als,
- Assistenzärztin/Assistenzarzt (Trainee) die Perspektive der Allgemeinen Inneren Medizin
- Ausbildner:in (Supervisor) die Perspektive der Palliative Care

Beschreiben Sie die Stärken des River-Aare-Curriculum

Bitte beschreiben Sie als,
- Assistenzärztin/Assistenzarzt (Trainee) die Perspektive der Allgemeinen Inneren Medizin
- Ausbildner:in (Supervisor) die Perspektive der Palliative Care

Welche Verbesserungsmöglichkeiten sehen Sie für das River-Aare-Curriculum

Bitte beschreiben Sie als,
- Assistenzärztin/Assistenzarzt (Trainee) die Perspektive der Allgemeinen Inneren Medizin
- Ausbildner:in (Supervisor) die Perspektive der Palliative Care

INSELSPITAL
UNIVERSITÄTSSPITAL BERN
HÔPITAL UNIVERSITAIRE DE BERNE

UNIVERSITÄRES ZENTRUM FÜR PALLIATIVE CARE

Rückmeldungen zum Kompetenzkatalog

Zur Verbesserung des Kompetenzkataloges würden wir gerne noch Ihr Feedback zum Kompetenzkatalog einholen.

Hier können Sie diesen nochmals einsehen.

PGI-lamapoll.pdf📎

Hier können Sie diesen nochmals einsehen.

Lamapoll-PGNS.pdf📎

Haben Sie allgemeine Kommentare zum Kompentenz-Katalog ?

Bitte beschreiben Sie als,
- Assistenzärztin/Assistenzarzt (Trainee) die Perspektive der Allgemeinen Inneren Medizin
- Ausbildner:in (Supervisor) die Perspektive der Palliative Care

Gibt es redundante Kompetenzen oder Kompetenzen welche für den Praxisalltag einer internistischen Fachperson nicht relevant sind??

Bitte beschreiben Sie als,
- Assistenzärztin/Assistenzarzt (Trainee) die Perspektive der Allgemeinen Inneren Medizin
- Ausbildner:in (Supervisor) die Perspektive der Palliative Care

Gibt es fehlende oder zu präzisierende Kompetenzen?

Bitte beschreiben Sie als,
- Assistenzärztin/Assistenzarzt (Trainee) die Perspektive der Allgemeinen Inneren Medizin
- Ausbildner:in (Supervisor) die Perspektive der Palliative Care

INSELSPITAL
UNIVERSITÄTSSPITAL BERN
HÔPITAL UNIVERSITAIRE DE BERNE

UNIVERSITÄRES ZENTRU
FÜR PALLIATIVE CARE

Wir danken ihnen herzlich für die Teilnahme

Möchten Sie über die Resultate dieser Umfrage informiert werden?

Bemerkung: Die E-mail Adresse wird unabhängig von den Antworten gespeichert. Die Anonymität wird also bewahrt.

Dann hinterlassen Sie bitte Ihre E-Mail-Adresse.

INSELSPITAL
UNIVERSITÄTSSPITAL BERN
HÔPITAL UNIVERSITAIRE DE BERNE
UNIVERSITÄRES ZENTRU
FÜR PALLIATIVE CARE

Wir danken Ihnen für Ihr Interesse

Dürften wir Sie fragen, wieso Sie die Umfrage nicht beantworten möchten?

Es ist Ihnen selbstverständlich freigestellt diese Frage zu beantworten. Sie würden uns damit helfen die Gültigkeit des Resultate zu verbessern

- [ ] Keine Zeit
- [ ] Kein Interesse an der Umfrage
- [ ] andere Gründe/möchte keine Gründe angeben

Umfrage erstellt mit

[illegible]
LamaPoll
