# Supplementary material for: Basic Training in Palliative Medicine for Internal Medicine Residents: Pilot Testing of a Canadian Model in Switzerland
Source: Palliat Med Rep. 2024 Apr 15;5(1):171–6. doi: 10.1089/pmr.2024.0004 (PMC11043622; doi:10.1089/pmr.2024.0004)
Supplement: Supplemental data [file Suppl_AppSA3-2.pdf]

INSELSPITAL
UNIVERSITÄTSSPITAL BERN
HÔPITAL UNIVERSITAIRE DE BERNE

UNIVERSITÄRES ZENTRL
FÜR PALLIATIVE CARE

Evaluation of the pilot phase of the "River Aare Curriculum"

Dear Colleagues

First of all, we would like to thank you for participating in the pilot phase of the River Aare curriculum at our station. We hope that some of what you have learned will be helpful for your future career.
In order to improve and adapt the curriculum to the needs of internists, we would also like to learn from you. That is why you are receiving a questionnaire from us today. It is short and takes about 10-15 minutes to complete.

We would be very pleased if we could have your feedback and thank you in advance for your interest.

Andreas Ebneter, for the project team

Project team:
- Andreas Ebneter; Steffen Eychmüller; Petra Mair; Barbara Affolter

Copyright: The project is based on a curriculum of the Canadian Society of Palliative Care Physicians (CSPCP) and the University of Toronto. The content is subject to copyright and may not be distributed/shared without permission. We would like to thank our colleagues, especially our international consultant (Prof. Ebru Kaya), for permission to use it and adapt it to the Swiss context.

Data protection: Your data will be anonymized (when entered into the online tool), encrypted and analyzed without drawing conclusions about individuals. By answering this survey, you agree that your answers (anonymized) may be used for publication.

Ethics/data protection: The survey is part of an accompanying quality improvement study, the cantonal ethics committee has reviewed the project plan and confirmed it as not requiring approval (quality assurance study, Req-2023-00646), the data protection rules are complied with in accordance with GCP, the online survey tool is certified in accordance with GDPR (Europe), the data is stored in Germany)

Do you agree to participate?

- ◯ Yes, I would like to participate
- ◯ No, rather not

INSELSPITAL
UNIVERSITÄTSSPITAL BERN
HÔPITAL UNIVERSITAIRE DE BERNE
UNIVERSITÄRES ZENTRUM
FÜR PALLIATIVE CARE

Evaluation of the "River Aare Curriculum"

My role in the clinic is...

- ◯ Assistant doctor (trainee) Trainer
- ◯ (supervisor)

My place of work

◯ University hospital ◯ Non-university hospital

How many years of experience do you have as a doctor?

[ ] years

Do you already have clinical experience in palliative care?

◯ Yes, following number of months [ ] ◯ No

How confident do you feel about caring forpatients with palliative care needs?

uncertain	rather uncertain	neither safe/nor uncertain	rather safe	safe
◯	◯	◯	◯	◯

What curriculum structure did you complete during your rotation on the palliative care ward?

◯ "Long" curriculum ◯ "short" curriculum

How many weeks were you on the palliative care ward?

[ ] Weeks

What curriculum structure does your assessment as a supervisor refer to?

◯ "Long" curriculum ◯ "short" curriculum

As a supervisor, are you evaluating the River Aare curriculum for the first time?

◯ Yes

◯ No, (please specify number of enter previous evaluations) [ ]

How would you rate the overall quality of the River Aare curriculum?

very bad	poor	average	good	very good
○	○	○	○	○

How do you assess the feasibility of the River Aare curriculum?

	very bad	poor	average	good	very good
With regard to the scope/amount of learning content	○	○	○	○	○
With regard to time resources	○	○	○	○	○

How would you rate the following aspects of the River Aare curriculum

	very bad	poor	average	good	very good
Organization of the site	○	○	○	○	○
Educational design	○	○	○	○	○
Educational support	○	○	○	○	○
Learning/teaching experience	○	○	○	○	○
Equipment/rooms (Facilities)	○	○	○	○	○

INSELSPITAL
UNIVERSITÄTSSPITAL BERN
HÔPITAL UNIVERSITAIRE DE BERNE

UNIVERSITÄRES ZENTRUM
FÜR PALLIATIVE CARE

Qualitative comments

In the next section you have the opportunity to send us individual comments. These may also be brief (keyword-like).

Comments on the general quality/practical relevance of the River Aare curriculum

Please describe as,
- Resident physician (trainee) the perspective of general internal medicine
- trainer (supervisor) the perspective of palliative care

Describe the strengths of the River Aare curriculum

Please describe as,
- Resident physician (trainee) the perspective of general internal medicine
- trainer (supervisor) the perspective of palliative care

What potential improvements do you see for the River Aare curriculum?

Please describe as,
- Resident physician (trainee) the perspective of general internal medicine
- trainer (supervisor) the perspective of palliative care

Feedback on the Syllabus (Competencies)

We would like to get your feedback on the skills catalog in order to improve it.

You can view it again here.

PGI-lamapoll.pdf

You can view it again here.

Lamapoll-PGNS.pdf

Do you have any general comments on the competence catalog?

Please describe as,
- Resident physician (trainee) the perspective of general internal medicine
- trainer (supervisor) the perspective of palliative care

Are there redundant competencies or competencies that are not relevant to the everyday practice of an internal medicine specialist?

Please describe as,
- Resident physician (trainee) the perspective of general internal medicine
- trainer (supervisor) the perspective of palliative care

Are there missing competencies or competencies that need to be specified?

Please describe as,
- Resident physician (trainee) the perspective of general internal medicine
- trainer (supervisor) the perspective of palliative care

INSELSPITAL
UNIVERSITÄTSSPITAL BERN
HÔPITAL UNIVERSITAIRE DE BERNE
UNIVERSITÄRES ZENTRUM
FÜR PALLIATIVE CARE

We would like to thank them for their participation

Would you like to be informed about the results of this survey?

Note: The e-mail address is stored independently of the answers. Anonymity is therefore preserved.

Then please leave your e-mail address.

Thank you for your interest

May we ask you why you do not wish to answer the survey?

You are of course free to answer this question. You would help us to improve the validity of the results

- [ ] No time
- [ ] No interest in the survey
- [ ] other reasons/would not like to give reasons

Survey created with
