# Supplementary material for: Basic Training in Palliative Medicine for Internal Medicine Residents: Pilot Testing of a Canadian Model in Switzerland
Source: Palliat Med Rep. 2024 Apr 15;5(1):171–6. doi: 10.1089/pmr.2024.0004 (PMC11043622; doi:10.1089/pmr.2024.0004)
Supplement: Supplemental data [file Suppl_AppSA4.docx]

Appendix 4: Detailed description of the setting (**Clinical Palliative Care Program)**

The university unit is part of the full-service (inpatient and outpatient service) University Center for palliative care, led by one director (professor, physician), a co-director (Ph.D., nurse), and one senior hospital physician. As for the unit (10 beds), one chief resident (fellow) supervises one resident. The resident, the targeted learner, is on rotation, usually 3–12 weeks, from the internal medicine department. Advanced residents (PGJ 3‒5/6) will typically be on this rotation. One physician and one nurse staff the inpatient consultation service, and the outpatient service is provided 1.5 days per week directly by a trained palliative care physician.

The non-university setting is two rural palliative care units, with the following structure.

One non-university palliative care unit has 11 patient beds and is part of the Department of Internal Medicine, which is managed by the head physician. The palliative care unit is staffed by an assistant doctor (1–2 resident year) and a specialist in palliative medicine. The consultation service, the background service (7/24), and the outpatient palliative medicine consultations (1 afternoon per week) are also covered by a specialist in palliative medicine.

The other non-university site is a smaller unit with 4-5 beds, embedded in the internal medicine department, led by one palliative care physician, supervising one resident. Residents rotating in the Department of Internal Medicine usually work in the unit for a few weeks. Outpatient service is provided one day per week by the palliative care physician.
