# Supplementary material for: Basic Training in Palliative Medicine for Internal Medicine Residents: Pilot Testing of a Canadian Model in Switzerland
Source: Palliat Med Rep. 2024 Apr 15;5(1):171–6. doi: 10.1089/pmr.2024.0004 (PMC11043622; doi:10.1089/pmr.2024.0004)
Supplement: Supplemental data [file Suppl_AppSA5.docx]

Appendix 4: Quotes of the participants

(Translation with deepl.com)

### General aspects of the curriculum

«Gute Qualität, hohe Relevanz für Allgemeiner Innere Medizin.»

"Good quality, high relevance for general internal medicine."

- Trainee-PGI-

“ich finde es ist gut, dass man trotz sehr kanpper zeitl. Ressourcen immer wieder daran erinnert wird, dass man noch gezieltes teaching machen kann/muss/soll»

*"I think it's good that, despite very limited time resources, you are constantly reminded that you can/must/should still do targeted teaching"*

- Supervisor-PGI-

«Die Aspekte, welche im Curriculum abgedeckt sind, sind für viele Bereiche in der Medizin anwendbar, insbes. in der Inneren Medizin. Gerade im Spitalalltag ist man auf internistischen Bettenstationen viel mit terminalen Erkrankungen konfrontiert.»

*«The aspects covered in the curriculum are applicable to many areas of medicine, especially internal medicine. Especially in everyday hospital life, you are often confronted with terminal illnesses on internal medicine wards.»*

- Trainee, PGNS-

“Mittels Curriculum erhalten die AA/AÄ einen sehr guten und relativ tiefen Einblick in die Palliative Care, welches aufgrund der bisher nur relativ kurzen Unterrichtseinheit im Studium aber bei gleichzeitig steigendem Bedarf an Palliativmedizin sehr sinnvoll ist.”

*"The curriculum gives the (residents) a very good and relatively deep insight into palliative care, which makes a lot of sense given the relatively short teaching unit in the degree course to date and the increasing need for palliative medicine."*

- Supervisor, PGNS-

### Strengths of the curriculum in general

*“Central palliative care topics covered.”*

- Trainee-PGI-

*“I think it's good that, despite very limited time resources, you are always reminded that you can/must/should still do targeted teaching”*

- Supervisor-PGI –

*“- The given structure is very helpful, I would also like to see it for other rotations*

*- Very detailed 1:1 further training*

*- enormous increase in knowledge (I have never acquired so much clinical knowledge in such a 'short' time)*

*- the great focus on recording symptoms + symptom control is very relevant to everyday life”*

- Trainee-PGNS-

*“The fact that you are doing the curriculum forces both supervisor and trainee to think about it again and again and to do the teaching despite limited time resources. Both sides benefit from this*-“

- Supervisor, PGNS-

### Suggested improvement of the curriculum in general

*“…… better information for the Chief resident about tasks and structured assessment with the assistant, more practice in implementation, currently still very new so not everything is completely clear yet…..”*

- *Trainee-PGI-*

*“….have not always managed to have access to the material*

*It is also difficult for me to keep track of the IT solution …”*

- *Supervisor-PGI-*

*“Stricter weekly focus to keep time management under control.”*

- Trainee, PGSN –

*“Time resources rather scarce*

*Documentation tool rather complicated”*

- Supervisor, PGNS -

## General comments - Specific for the competencies

Postgraduate immersion curriculum (PGI)

*«…. sometimes difficult to assess as competence in medicine is not the same as in palliative care …..)»*

*-Trainee-*

*«Short and concise catalog of skills in the short curriculum»*

-Supervisor -

Postgraduate non-specialist curriculum (PGNS)

“Very detailed, certain 'soft' points could be omitted if necessary (see below) “

- Resident-

“Discussion of symptoms and symptom control requires a lot of time and multiple 'sessions', could be divided into several blocks of individual symptoms or symptom complexes”

- supervisor-

- Spiritual care is addressed very extensively

“Perhaps communicating with dying patients/bringing bad news”

- Supervisor-

## Relevant redundancies with internal medicine training — Specific for the competencies.

Postgraduate immersion curriculum (PGI)

*none for trainees*

*“not really noticed yet”*

- Supervisor-

Postgraduate non-specialist curriculum (PGNS)

*“ Competencies about social/spiritual problems could still be summarized, but are not always easy to record with workplace-based assessment and are partly absorbed in everyday ward work”*

- Trainee –

*“The block on Suffering in Patients and Families and the communication block could be shortened and summarized.”*

- Trainee-

*“Spiritual care is addressed very extensively”*

- Supervisor-

## Missing items — Specific for the competencies.

Postgraduate immersion curriculum (PGI)

*None from trainees –*

«….miss communication, as an explicit part of teaching»

- Supervisor –

Postgraduate non-specialist curriculum (PGNS)

*None from trainees*

*«perhaps communicating with dying patients/bringing bad news»*

- Supervisor-
